# Postharvest Stability of Peeled Garlic Cloves (*Allium sativum L.*): A Study on Packaging Materials and Shelf‐Life Environmental Interactions

**DOI:** 10.1002/fsn3.71170

**Published:** 2025-11-10

**Authors:** Hany S. El‐Mesery, Mansuur Husein, Zicheng Hu, Mona A. Elabd

**Affiliations:** ^1^ School of Energy and Power Engineering Jiangsu University Zhenjiang China; ^2^ Agricultural Engineering Research Institute Agricultural Research Center Dokki, Giza Egypt; ^3^ School of Environment and Safety Engineering Jiangsu University Zhenjiang China; ^4^ Department of Water and Environmental Engineering, Faculty of Engineering Tamale Technical University Tamale Ghana; ^5^ Food Engineering and Packaging Department Food Technology Research Institute, Agriculture Research Center Dokki, Giza Egypt

**Keywords:** garlic, packaging material storage conditions, physicochemical, phytochemical, storage

## Abstract

This study investigates the influence of storage environments and packaging materials on peeled garlic's physicochemical, phytochemical, and microbiological stability (*Allium sativum L*.) during a 30‐day storage period. Four packaging materials, waxed paper (WP.B.), perforated low‐density polyethylene (P‐LDPE), low‐density polyethylene (LDPE), and mesh bags (M.B.), were evaluated under two storage conditions: refrigeration at 4°C and ambient temperature at 25°C. A comprehensive set of quality indicators was monitored, including firmness, moisture content, total soluble solids (TSS), titratable acidity (TA), pH, color change, pyruvic acid, allicin content, and microbial counts. Results revealed that storage temperature was the dominant factor influencing quality degradation. Peeled garlic stored at 25°C exhibited rapid declines in firmness, allicin, and pyruvic acid, alongside significant increases in weight loss, color change, and microbial loads. In contrast, refrigerated storage significantly slowed deterioration, particularly when combined with breathable packaging materials like (WP.B.) and (P‐LDPE), which maintained higher levels of bioactive compounds and reduced microbial proliferation. Correlation matrix analysis showed strong negative correlations between storage time and key quality parameters (firmness: *r* = −0.981, allicin: *r* = −0.999), while principal component analysis (PCA) confirmed the clustering of spoilage‐related variables along PC1 under ambient conditions. These findings highlight the need for targeted storage strategies and the development of advanced, intelligent packaging technologies. The study recommends modeling degradation kinetics, exploring thermodynamic parameters for bioactive stability, and integrating multi‐topic microbial profiling for predictive quality control. This approach will enable more sustainable, science‐driven garlic storage solutions tailored to diverse postharvest supply chains.

## Graphical abstract

The influence of storage environments and packaging materials on garlic's physicochemical, phytochemical, and microbiological stability (*Allium sativum L.*) during a 30‐day storage period. Four packaging materials, waxed paper (WP.B), perforated low‐density polyethylene (P‐LDPE), low‐density polyethylene (LDPE), and mesh bags (M.B), were evaluated under two storage conditions: refrigeration at 4°C and ambient temperature at 25°C. These findings highlight the need for targeted storage strategies and the development of advanced, intelligent packaging technologies. The study recommends modeling degradation kinetics, exploring thermodynamic parameters for bioactive stability, and integrating multi‐topic microbial profiling for predictive quality control. This approach will enable more sustainable, science‐driven garlic storage solutions tailored to diverse postharvest supply chains.

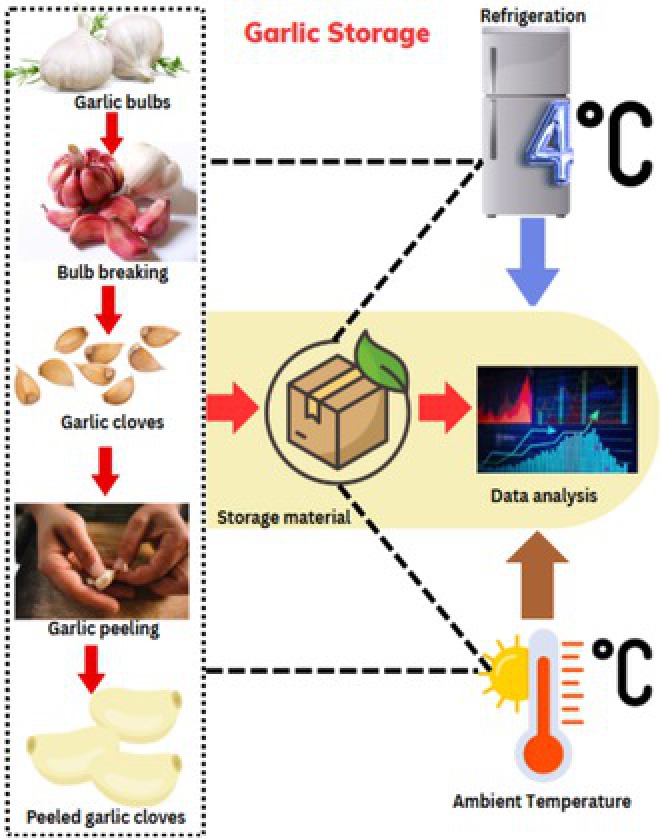

## 1 Introduction

Garlic (*Allium sativum L*.) is among the most commonly used culinary and medicinal plants, celebrated for its unique flavor and health benefits. It possesses various bioactive components, including organosulfur compounds (such as allicin), phenolic acids, flavonoids, and antioxidants, all contributing to its antimicrobial, anti‐inflammatory, anticancer, and cardioprotective effects (Bayan et al. 2014; Feng et al. 2021). Due to its widespread application in food and pharmaceutical sectors, preserving garlic quality during postharvest storage is vital for maintaining its nutritional and functional attributes. However, garlic is subject to rapid perishing and is susceptible to physiological and biochemical decline, which includes moisture loss, sprouting, microbial spoilage, and a decrease in bioactive components (El‐Mesery, Qenawy, et al. 2025). Thus, it is essential to optimize storage conditions and packaging materials to prolong shelf life while safeguarding its physicochemical and phytochemical properties (Hong et al. 2001; Hu et al. 2017). Among these, pyruvic acid plays a crucial role as an indicator of garlic pungency and enzymatic activity. It reflects alliinase‐mediated hydrolysis of alliin, which is fundamental to the formation of allicin and related sulfur compounds responsible for garlic's flavor and health‐promoting properties (Feng et al. 2018; González et al. 2013).

Postharvest losses in garlic mainly occur due to inadequate storage conditions, leading to weight loss, sprouting, mold, and nutrient loss. Proper garlic preservation is crucial for maintaining its sensory qualities and health‐promoting compounds. Research indicates that storage temperature, humidity, and atmospheric composition significantly impact garlic's shelf life (Cantwell et al. 2003; Lin et al. 2018). For example, keeping garlic at low temperatures (0°C–5°C) can inhibit sprouting and microbial growth, while high moisture levels encourage fungal contamination. Appropriate packaging materials can also minimize moisture loss and gas exchange, enhancing garlic's quality over time (Huang, Ding, et al. 2018; Huang, Pan, et al. 2018; Tavar et al. 2024).

Packaging protects garlic from environmental elements, including oxygen, light, and humidity. Conventional packaging materials such as polyethylene and polypropylene have been widely tested for peeled garlic storage, while breathable alternatives like waxed paper and mesh bags are also investigated for their effects on quality retention (He et al. 2019; Huang, Ding, et al. 2018; Huang, Pan, et al. 2018). Active packaging that contains antimicrobial agents shows promise in further enhancing garlic preservation by preventing microbial growth and oxidative reactions. Furthermore, biodegradable materials like polylactic acid (PLA) and chitosan‐based films are increasingly recognized for their environmental benefits and efficacy in preserving garlic quality (El‐Mesery, Omran, et al. 2025; El‐Mesery, Qenawy, et al. 2024).

Storage conditions, such as temperature, relative humidity (RH), and light exposure, significantly affect garlic's postharvest physiology. Refrigeration (0°C–4°C) is often employed to prolong shelf life by minimizing metabolic activity; however, inadequate humidity control may result in condensation and mold growth (EL‐Mesery et al. 2022; Shagun et al. 2024). Although storing at room temperature is cost‐effective, it promotes sprouting and biochemical degradation without proper packaging. Research indicates that a blend of low temperature (0°C–2°C) and high RH (65%–75%) can significantly decrease weight loss and sprouting in garlic (Cui et al. 2019; Yoosefian et al. 2023). Moreover, non‐thermal preservation methods like gamma irradiation and ozone treatment have been examined to prevent sprouting and microbial growth while preserving nutritional quality (EL‐Mesery et al. 2022).

On the other hand, low humidity helps minimize excess moisture on the garlic's surface, thus reducing the likelihood of rot and extending its shelf life. As people become more health‐conscious, interest in garlic is growing due to its substantial nutritional benefits and extended shelf life. However, achieving these goals presents challenges, such as identifying ideal storage environments and choosing suitable packaging products to preserve garlic's physicochemical properties (El‐Mesery, Adelusi, et al. 2024). This study highlights the critical role of packaging and storage conditions in preserving garlic. It aims to evaluate how different shelf‐life environments and packaging materials influence garlic's physicochemical and phytochemical properties. By identifying the most effective storage and packaging techniques, this research seeks to minimize postharvest losses and enhance the commercial value of garlic.

## 2 Materials and Methods

### 2.1 Garlic Preparation

The study used the locally grown cultivar “Yunnan White,” harvested at physiological maturity guaranteeing consistency in size, maturity, and visual quality. After harvest, bulbs were cured for 7 days under shade with natural ventilation, before being transported to the laboratory for further processing. Bulbs exhibiting indications of mechanical damage, disease, or physiological abnormalities were removed from consideration. The chosen samples were meticulously cleaned using distilled water to eliminate soil and debris. The clove was peeled using a garlic peeler machine. Garlic cloves were treated with a sodium hypochlorite solution (200 ppm) for 2 min to decrease the initial microbial load. After that, they were rinsed thoroughly with sterile distilled water to eliminate leftover chlorine. The garlic cloves were stored in a laboratory at 4°C before application. After meticulously examining the garlic, those damaged or rotten cloves were removed.

### 2.2 Packaging Materials

Garlic cloves were classified into four distinct packaging treatments according to their physical condition and the materials used: (i) waxed paper bag (WP.B), (ii) low‐density polyethylene (LDPE), (iii) perforated low‐density polyethylene (P‐LDPE), and (iv) mesh bags (M.B). Various packaging materials were used to weigh the garlic cloves at 300 g.

### 2.3 Shelf‐Life Conditions

The packaging treatments were systematically divided for storage at two distinct temperature conditions: 4°C (cold storage) and 25°C (ambient storage), resulting in a comprehensive total of eight treatment combinations. Ambient storage was conducted in a laboratory with a temperature range from 24°C–26°C, with an average of 25°C. For clarity, the mean value of 25°C is reported throughout the manuscript. The garlic's physicochemical characteristics were evaluated every 0–5 days for 30 days. Each treatment consisted of three independent replicates, with 300 g of garlic cloves per replicate. All physicochemical, biochemical, and microbiological analyses were performed in triplicate per replicate to ensure statistical robustness.

### 2.4 Determination of Total Soluble Solids (TSS), Titratable Acids (TA), and pH

Garlic juice samples were obtained by blending 10 g of garlic cloves with 50 mL of distilled water using a homogenizer (Ultra‐Turrax, IKA, Germany) at 12,000 rpm for 2 min at 4°C. The homogenate was then filtered through Whatman No. 1 filter paper, and the clear extract was used for physicochemical analyses. The measurements included total soluble solids (TSS), titratable acids (TA), and pH. TSS was measured at 20°C using a handheld refractometer made by ATAGO (Tokyo, Japan). pH was measured with a calibrated digital pH meter (Mettler Toledo, USA) at 25°C. Titratable acidity was determined by mixing 10 g of the homogenate with 100 mL of water, followed by titration with 0.1 mol/L of Sodium Hydroxide (NaOH), and expressed as citric acid equivalents, as citric acid is a commonly used reference for acidity in food products. The TA is represented as the percentage of citric acid, and pH was also measured using a potentiometer set to 25°C (Prakash and Prasad 2023).

### 2.5 Analysis of Moisture Content and Weight Loss

The garlic's moisture percentage was assessed using the microwave technique (AOAC 2005). A 20 g piece of garlic was based on a pre‐dried aluminum sheet for 4 h at a temperature of 105°C. Several factors, both before and after harvesting, affect fruit weight reduction, including the timing of the harvest and storage temperature. Garlic quality can be indicated by weight loss, as a significant decrease results in the tissue becoming soft and dull. The garlic's moisture content (MC) was determined using Equation (1) (Liu et al. 2025).

(1) $$\mathrm{Mc}=\frac{W_{i}-W_{d}}{W_{d}}$$

*W*_*d*_ and *W*_*i*_ denote the garlic's dried and initial weights, respectively.

A durable electrical weighing balance was utilized to assess the garlic's mass at the start of the experiment and at various time intervals throughout the storage period to measure the garlic's weight loss. The results were reported as a percentage of the initial weight loss, following Equation (2).

(2) $$\text{Weight Loss}=\frac{\text{Initial weight}-\text{Final weight}}{\text{Final weight}}\times 100\%$$

### 2.6 Firmness Determination

Firmness is essential for evaluating garlic quality and influencing a buyer's choice when selecting freshly harvested garlic. It is a key texture parameter for assessing a material's mechanical resistance when subjected to an applied force. Commonly evaluated in food, pharmaceuticals, cosmetics, and industrial materials, firmness testing helps determine product quality, consistency, and usability. After removing the garlic cloves from the storage compartment, they were processed into uniform samples using a double cutter and punch, measuring 15 mm in diameter and 10 mm in thickness. The Texture Profile Analysis (TPA) mode was employed during testing at room temperature (25°C) with a texture analyzer (Stable Micro System TA‐XTPL, UK). The test conditions were as follows: the sample block underwent two compression processes utilizing a type P‐25 cylindrical probe (He et al. 2019). The pretest speed was set at 0.1 cm/s, the test speed at 0.5 cm/s, and the post‐test speed at 0.1 cm/s. The strain was 60%, and the trigger force was 5 g, with the test conducted over 5 replicates.

### 2.7 Color Measurement

A movable colorimeter (CR‐400 Chroma Meter, Konica Minolta, USA) was utilized to measure the color variation of garlic. Following the calibration with a standard white plate, five assessments were conducted on the four samples across the various storage conditions. Triple measurements were employed to acquire the *L*, *a*, and *b* values. The total variation in color (𝛿E) was calculated using Equation (3) to analyze the sample's color variation compared to the control.

(3) $$\delta E=\sqrt{\;{\left(L^{*}-L_{0}^{*}\right)}^{2}+{\left(a^{*}-a_{0}^{*}\right)}^{2}+{\left(b^{*}-b_{0}^{*}\right)}^{2}}$$

where *a*, *b*, and *L* represent redness/greenness values, yellowness/blueness, and whiteness, respectively. The color value of the garlic is denoted by the subscript “0”. When the value of 𝛿E is high, it signifies a larger change in the color of the blanched sample.

### 2.8 Determination of Bioactive Compounds

The stability and retention of garlic's bioactive compounds were investigated by quantitatively determining allicin and pyruvic acid. Allicin content (mg/g fresh weight) was measured using high‐performance liquid chromatography (HPLC) equipped with a UV detector set at 254 nm (El‐Mesery, Qenawy, et al. 2024). Pyruvic acid concentration (μmol/g) was assessed via the 2,4‐dinitrophenylhydrazine (DNPH) colorimetric method, which reflects garlic pungency and indicates alliinase activity. For allicin determination, 5 g of garlic cloves was homogenized in 20 mL methanol, centrifuged at 5000 rpm for 10 min, and the supernatant was filtered (0.45 μm) prior to HPLC analysis. For pyruvic acid, 2 g of homogenized garlic tissue was reacted with DNPH reagent, and absorbance was measured at 420 nm following standard protocols (Wongsa et al. 2023).

### 2.9 Microbiological Assessments

Microbial quality was determined by enumerating total aerobic mesophilic bacteria (AMC), fungi, and yeast populations. Microbial counts were obtained by homogenizing 10 g of the garlic sample in 90 mL sterile peptone water and performing serial dilutions. Samples were plated on Plate Count Agar (PCA) for AMC, Potato Dextrose Agar (PDA) for fungi, and Yeast Extract Glucose Chloramphenicol Agar (YGC) for yeasts. Plates were incubated at 30°C for 48–72 h, and results were recorded as log colony‐forming units per gram (log CFU/g).

### 2.10 Statistical and Multivariate Analysis

All experiments were conducted in triplicate, and data were presented as mean ± standard deviation. Statistical analysis was performed using OriginPro (2024). Pearson correlation coefficients were computed to determine relationships among physicochemical, biochemical, and microbiological variables. Principal Component Analysis (PCA) was conducted using OriginPro 2024 software to assess the multidimensional relationships among variables and identify significant quality change patterns under different storage environments.

## 3 Results and Discussion

The impact of storage temperature and packaging material on garlic's physicochemical and microbiological stability was systematically evaluated over 30 days. The results revealed distinct degradation trends influenced by environmental conditions and packaging types, providing critical insights into postharvest handling strategies for maintaining garlic quality.

### 3.1 Analysis of Firmness

**FIGURE 1:**
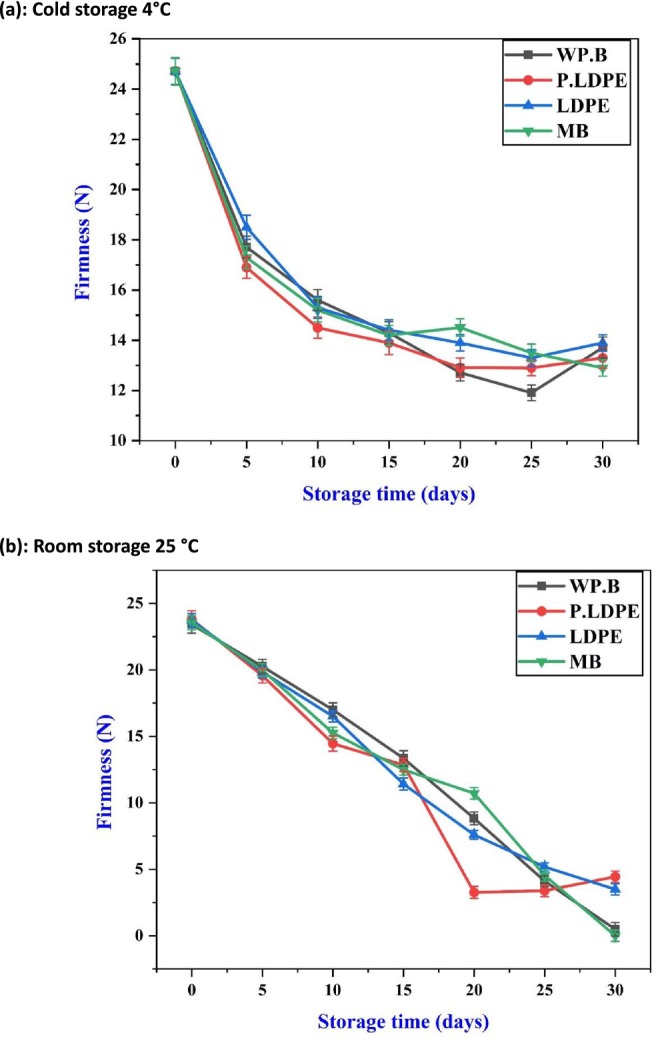
Effects of packaging materials and storage temperatures (a) cold storage 4°C, (b) room storage 25 °C on garlic cloves’ firmness. (i) waxed paper bag (WP.B), (ii) low‐density polyethylene (LDPE), (iii) perforated low‐density polyethylene (P‐LDPE), and (iv) mesh bags (M.B).

Figure 1 illustrates a comprehensive comparative examination of how different packaging materials and storage temperatures influence garlic's firmness throughout a 30‐day duration. Figure 1a illustrates garlic maintained at a refrigeration temperature of 4°C, whereas Figure 1b depicts garlic kept under ambient conditions at 25°C. Four types of packaging materials were assessed: (i) waxed paper bag (WP.B.), (ii) low‐density polyethylene (LDPE), (iii) perforated low‐density polyethylene (P‐LDPE), and (iv) mesh bags (M.B.). These results are crucial in understanding the impact of postharvest handling strategies on the physicochemical stability of garlic, offering essential insights for enhancing shelf life and maintaining quality retention. Under refrigerated storage (4°C), firmness exhibited a gradual decline across all treatments, with (WP.B.) and (M.B.) consistently showing higher firmness levels throughout the storage period in comparison to (P‐LDPE) and (LDPE). This indicates that breathable, natural materials like waxed paper and paper bags are superior in reducing moisture‐related textural degradation in calmer settings. The results are consistent with the work of (Madhu et al. 2021), who highlighted that semi‐permeable packaging materials can regulate internal humidity and gas exchange, consequently delaying enzymatic softening processes. The exceptional performance of (WP.B.) can be attributed to its capacity to maintain moisture retention while ensuring sufficient ventilation, thereby reducing microbial growth and cellular degradation. Furthermore, Figure 1a indicates that at 4°C, there was a notable decrease in weight loss for all types of packaging. Interestingly, (LDPE) and (P‐LDPE) demonstrated marginally superior moisture retention compared to (WP.B) and (M.B), potentially attributable to the reduced water vapor permeability inherent in polyethylene‐based materials. This finding is corroborated by (Liu et al. 2017), who indicated that synthetic films exhibiting high barrier properties significantly minimize water loss during cold storage. Nonetheless, this advantage might be accompanied by a compromise in textural integrity, as illustrated in Figure 1a, where (P‐LDPE) and (LDPE) are associated with reduced firmness values (Jiang et al. 2021a, 2021b).

Conversely, when stored at ambient conditions of 25°C (Figure 1b), there was a notable and consistent decrease in firmness across all treatments, especially marked by a swift decline observed between days 10 and 25. Perforated low‐density polyethylene (P‐LDPE) exhibited the most significant loss of firmness, highlighting the constraints of perforated polymer films under elevated temperatures. This pattern supports the findings of (Ding et al. 2020), who observed that polyethylene packaging frequently does not manage moisture properly in elevated temperature settings, leading to increased tissue dehydration and texture deterioration.

### 3.2 Dynamic Changes in Weight Loss and Moisture Content

**FIGURE 2:**
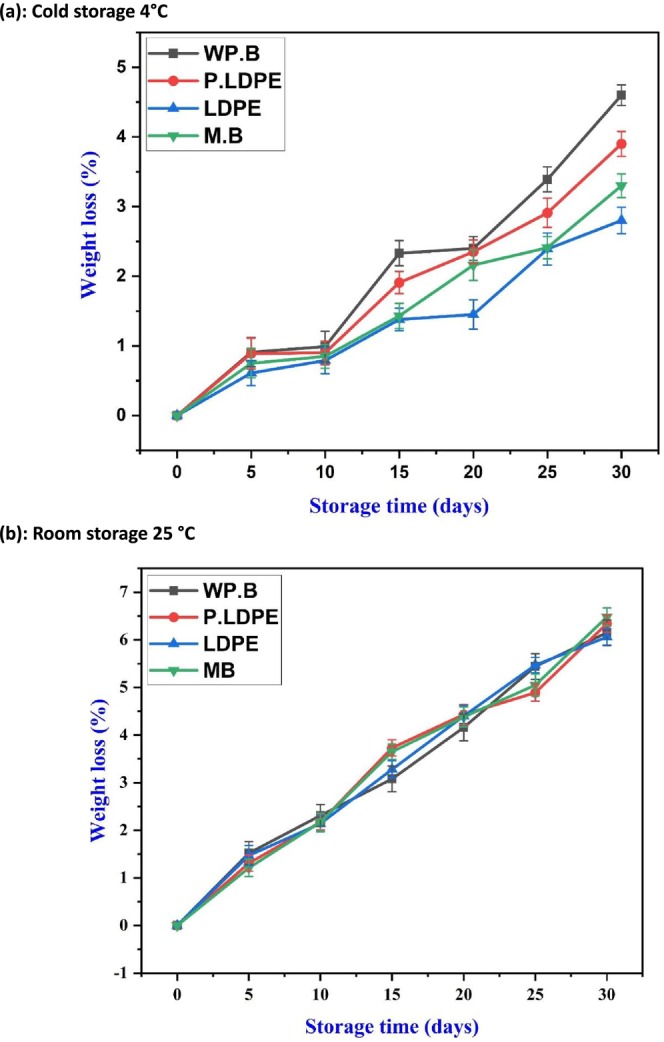
Effects of packaging materials and storage temperatures (a) cold storage 4°C, (b) room storage 25 °C on garlic cloves’ weight loss.(i) waxed paper bag (WP.B), (ii) low‐density polyethylene (LDPE), (iii) perforated low‐density polyethylene (P‐LDPE), and (iv) mesh bags (M.B).

The weight loss observed under different conditions (Figure 2) exhibited a linear trend, notably greater than that under refrigerated storage. Although the variations among packaging treatments were not highly pronounced, (WP.B.) and (LDPE) demonstrated a slight advantage over the others in minimizing weight loss. This indicates that as temperatures increase, the influence of packaging on moisture retention decreases, making temperature the primary factor affecting water loss. (EL‐Mesery et al. 2021) highlighted the impact of increased temperatures on enhancing respiration and transpiration rates in *allium* species, consequently intensifying desiccation. The findings emphasize the relationship between packaging material and storage temperature in preserving garlic after harvest. Refrigerated storage at 4°C (Figure 2a) significantly improves the efficacy of packaging methods, with waxed paper and paper bags proving the most effective in maintaining firmness. At the same time, high‐density polyethylene effectively reduces moisture loss. In contrast, when stored at ambient temperatures (25°C), the protective benefits of packaging diminish, indicating that active cooling is crucial for prolonging the shelf life of garlic (Figure 2b).

**FIGURE 3:**
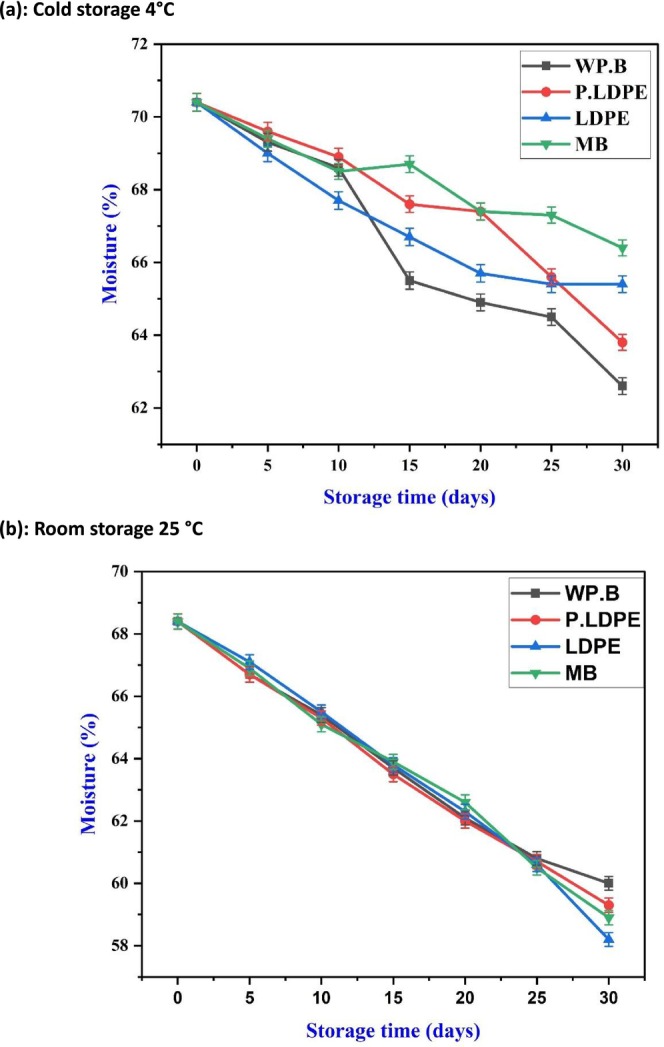
Effects of packaging materials and storage temperatures (a) cold storage, 4°C, (b) room storage, 25 °C, on garlic cloves’ moisture content. (i) waxed paper bag (WP.B), (ii) low‐density polyethylene (LDPE), (iii) perforated low‐density polyethylene (P‐LDPE), and (iv) mesh bags (M.B).

Moisture content at 4°C demonstrates a steady decrease across all packaging types as seen in Figure 3a. It is noteworthy that (P‐LDPE) and (LDPE) maintained elevated moisture levels throughout the majority of the storage duration, thereby validating the anticipated efficacy of polyethylene‐based materials as practical moisture barriers. Nonetheless, moisture retention may not always yield positive outcomes, as increased humidity within packaging could make bulbs more susceptible to microbial growth, particularly when exposed to minor temperature variations. This presents a crucial advancement, as optimizing moisture retention is preferable to maximizing it, which is attracting growing significance in the study of modified atmosphere packaging (MAP). Furthermore, (WP.B) exhibited the highest moisture loss, demonstrating enhanced breathability while also implying a heightened vulnerability to drying out. This may prove beneficial in reducing microbial risks during refrigerated storage, aligning with the observations made by (Wongsa et al. 2023; Zhao et al. 2023), who reported diminished decay in garlic kept at lower relative humidity. Mesh bags (M.B) also exhibited a comparable yet somewhat muted trend, reinforcing their viability as a balanced packaging solution in cool storage conditions. Under ambient storage conditions at 25°C, Figure 3b illustrates a significant and linear decrease in moisture content, regardless of the type of packaging used. This highlights the primary role of ambient temperature in water loss, overshadowing the effects of packaging (Guo et al. 2020; Jiang et al. 2021).

### 3.3 Dynamic Changes in Total Soluble Solids (TSS), Titratable Acidity (TA), and pH During Garlic Storage

**FIGURE 4:**
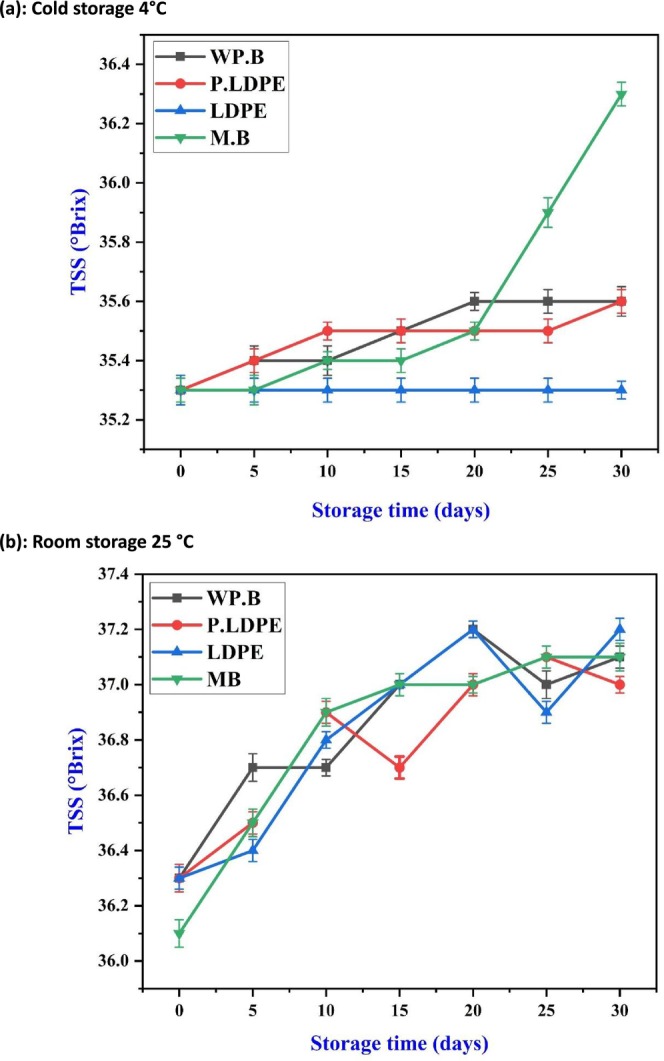
Effects of packaging materials and storage temperatures (a) cold storage 4°C, (b) room storage 25 °C on garlic cloves’ total soluble solids (TSS). (i) waxed paper bag (WP.B), (ii) low‐density polyethylene (LDPE), (iii) perforated low‐density polyethylene (P‐LDPE), and (iv) mesh bags (M.B).

**FIGURE 5:**
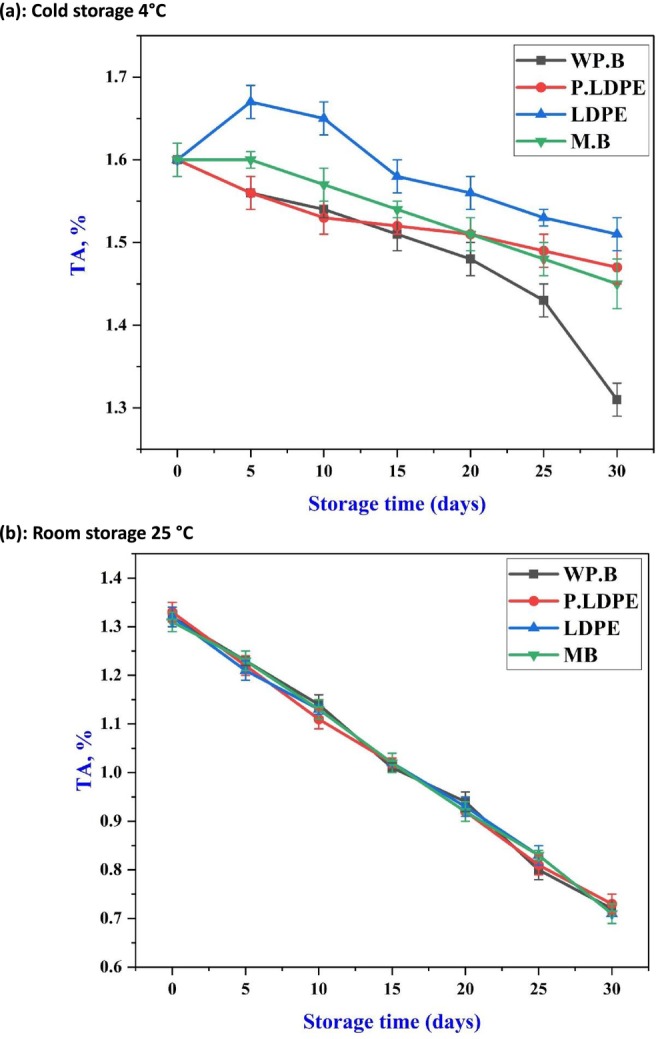
Effects of packaging materials and storage temperatures (a) cold storage 4°C, (b) room storage 25 °C on garlic cloves’ titratable acidity (TA). (i) waxed paper bag (WP.B), (ii) low‐density polyethylene (LDPE), (iii) perforated low‐density polyethylene (P‐LDPE), and (iv) mesh bags (M.B).

Figures 4 and 5 show variations in TSS and TA of garlic over a 30‐day storage period, exploring temperature conditions and packaging materials. Empirically, under cold storage (4°C), the TSS levels (Figure 4a) exhibited a relatively modest yet noticeable increase across all packaging materials, with the most significant accumulation noted in garlic stored in mesh bags (M.B), particularly after day 20. This pattern could indicate a gradual but ongoing breakdown of complex carbohydrates into simpler sugars, occurring under conditions of diminished metabolic activity. The results align with the work of (Singh 2018), who showed that cool storage improves carbohydrate preservation and may promote slow enzymatic breakdown, especially when breathable packaging like paper bags is utilized to prevent anaerobic stress. Interestingly, LDPE showed the smallest rise in TSS, indicating that moisture‐impermeable packaging might limit the mobilization of respiratory substrates because of inadequate gas exchange.

Simultaneously, titratable acidity (Figure 5) exhibited a gradual decline across all treatments, with the most notable reduction recorded in (WP.B) by day 30. The gradual decrease observed is typical of the catabolism of organic acids during respiration, a pattern influenced at lower temperatures by diminished enzymatic activity. The varying effectiveness of packaging materials in this context underscores the ability of waxed paper to promote mild dehydration and increased respiration, resulting in a quicker loss of acid relative to packaging that retains moisture more effectively. The results align with the findings of (Sailah et al. 2024), which indicated that semi‐permeable packaging materials in cold conditions can enhance acid depletion as a result of increased oxygen permeability.

Moreover, Figure 5b validates a steady and linear decrease in TA across all packaging types when stored at 25°C. The closely aligned trends observed across treatments show that temperature plays a more significant role in acid catabolism than packaging. This finding supports the conclusions drawn by He et al. (2019), about the preeminence of thermal factors in influencing respiratory‐driven chemical alterations in garlic. Although there are slight variations among packaging types, these differences are not substantial enough to meaningfully impact the trajectory of TA, highlighting the dominant influence of increased temperature on the depletion of organic acid reserves (Al‐Maqtari et al. 2021; Yan et al. 2021).

This research outcome highlights the essential relationship between packaging materials and storage temperature in influencing the biochemical quality of garlic. At 4°C, breathable packaging such as paper bags facilitates TSS accumulation and regulates TA decline, which may improve sensory quality. At 25°C, the advantages of packaging are significantly diminished due to swift metabolic degradation, highlighting the importance of cold storage to maintain the phytochemical integrity of garlic.

**FIGURE 6:**
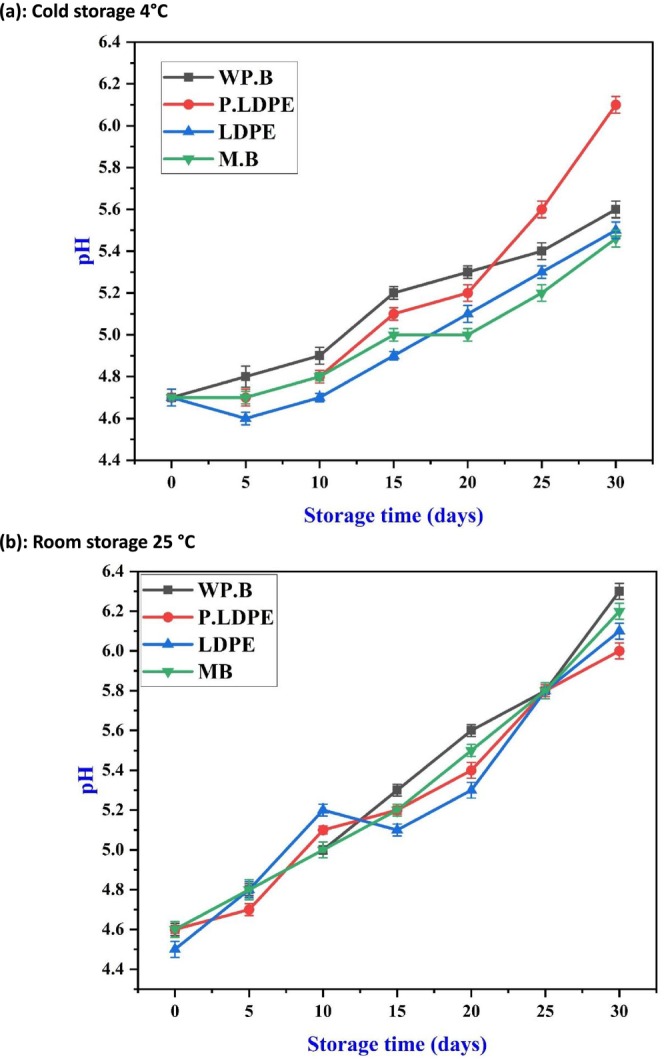
Effects of packaging materials and storage temperatures (a) cold storage 4°C, (b) room storage 25 °C on garlic cloves’ pH. (i) waxed paper bag (WP.B), (ii) low‐density polyethylene (LDPE), (iii) perforated low‐density polyethylene (P‐LDPE), and (iv) mesh bags (M.B).

Figure 6a shows that during refrigerated storage at 4°C, the pH of garlic revealed a gradual increase over time across all packaging materials, with the most significant rise noted in (P‐LDPE). This trend appears to indicate a slow neutralization of acidic compounds, possibly resulting from the depletion of organic acids through respiration and the partial loss of volatile sulfur compounds. It is noteworthy that the more rapid pH increase observed in (P‐LDPE) may suggest a higher metabolic turnover, potentially resulting from less effective regulation of internal humidity and oxygen levels. This trend was similarly observed by He et al. (2019) who indicated that inadequate packaging permeability could enhance oxidative processes and enzymatic breakdown in garlic, leading to an increase in pH levels (Lu et al. 2022; Zhou et al. 2021).

In contrast, (WP.B) and (M.B) demonstrated a more effective moderation of the pH shift. The various packaging types, exhibiting enhanced moisture‐buffering and optimal gas‐exchange balance, could contribute to prolonged metabolic homeostasis. The limited increase in pH observed in (LDPE) aligns with this observation. However, the elevated humidity present in sealed synthetic films may create slightly anaerobic conditions, which could slow down acid metabolism at first, but might later promote spoilage during storage (Gonzalles et al. 2021; Lin et al. 2021).

At ambient temperature (Figure 6b), the pH exhibited a more uniform increase across treatments, showing a nearly linear progression from day 0 to day 30. This indicates that higher temperatures speed up metabolic breakdown universally, reducing the varying impact of packaging materials. Nonetheless, (WP.B) and (M.B) demonstrated somewhat more alleviated increases, suggesting a potential for enhanced retention of organic acids or a more gradual breakdown of sulfurous compounds in drier microenvironments. The results align with the research conducted by (Ghidini et al. 2024; Hou et al. 2025), highlighting how temperature‐induced enzyme activation influences the conversion of pyruvate and sulfur compounds, which in turn affects pH trajectories in allium species (Hu et al. 2022; Zhang et al. 2022).

### 3.4 Analysis of Color Variation

**FIGURE 7:**
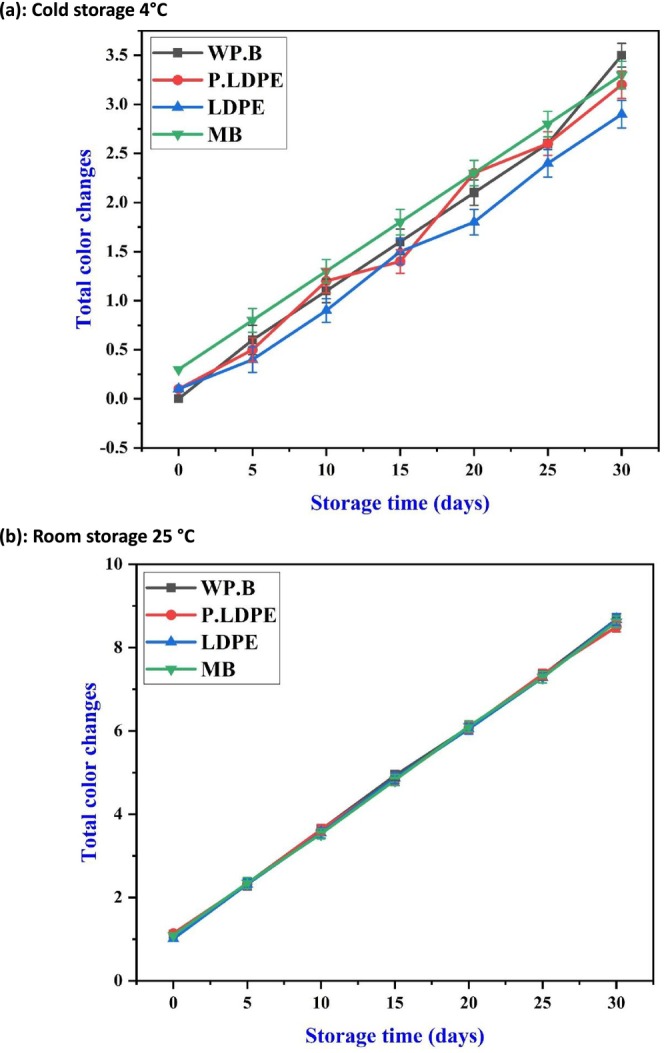
Effects of packaging materials and storage temperatures (a) cold storage 4°C, (b) room storage 25 °C on garlic cloves’ total color changes. (i) waxed paper bag (WP.B), (ii) low‐density polyethylene (LDPE), (iii) perforated low‐density polyethylene (P‐LDPE), and (iv) mesh bags (M.B).

Essential color changes have been systematically examined in Figure 7, which depicts 30 days of storage under different temperature conditions across four types of packaging materials. In the refrigerated conditions (Figure 7a), color values exhibited a gradual increase over time, with the most minimal color alteration noted in garlic preserved in (P‐LDPE) and (LDPE). This highlights packaging materials made from synthetic polymers, especially those designed to restrict oxygen and moisture transfer, that can significantly reduce enzymatic browning and pigment loss, which is one of the frequent challenges encountered after harvesting garlic. Nonetheless, the rise was maintained within acceptable visual limits throughout the storage period, emphasizing the beneficial role of low temperatures in postponing nonenzymatic browning.

Consistent with the findings of (Duan et al. 2022; Madhu et al. 2021; Misra and Roopesh 2019) the decrease in pigment degradation at lower temperatures can be linked to the inhibition of polyphenol oxidase (PPO) activity and a reduction in free radical generation. The interaction between temperature and packaging is clearly demonstrated in this instance, with breathable materials at lower temperatures effectively managing discoloration.

Under ambient conditions (Figure 7b), clove color changes exhibited a significant linear increase across all packaging types, displaying nearly identical trends. This indicates that increased temperature is the primary factor contributing to visual deterioration, surpassing the protective abilities of any individual packaging type. This highlights a crucial finding emphasizing that, thermal stress serves as the main factor influencing color stability in garlic, diminishing the relative advantages of packaging in ambient conditions. This underscores the necessity for innovative technologies such as UV‐filter films or antioxidant‐infused layers in packaging for shelf‐stable products.

### 3.5 Analysis of Allicin Content

**FIGURE 8:**
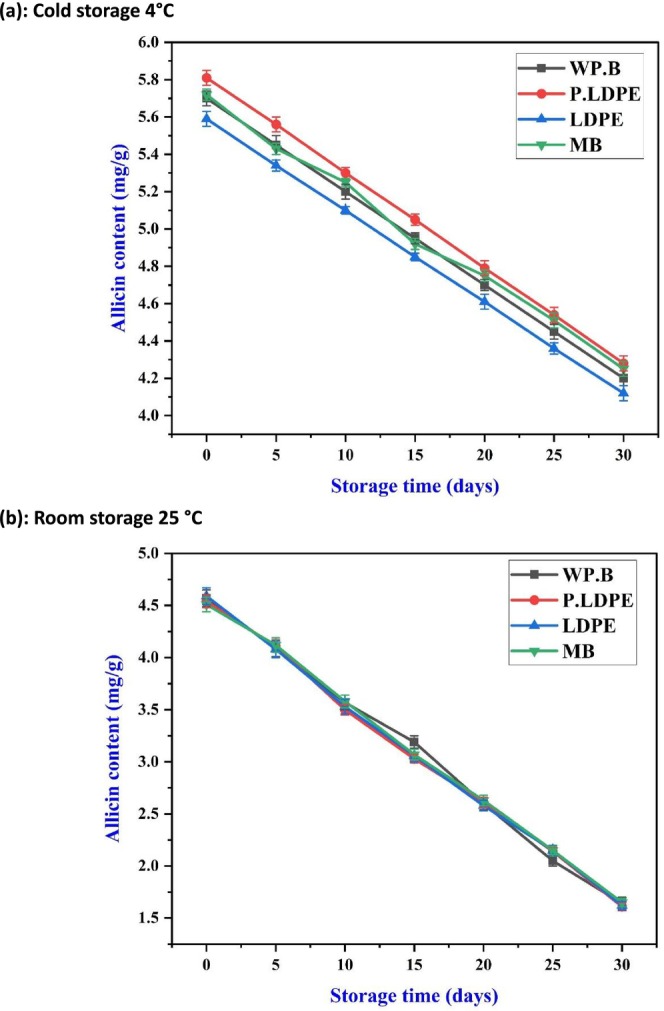
Effects of packaging materials and storage temperatures (a) cold storage 4°C, (b) room storage 25 °C on garlic cloves’ Allicin content. (i) waxed paper bag (WP.B), (ii) low‐density polyethylene (LDPE), (iii) perforated low‐density polyethylene (P‐LDPE), and (iv) mesh bags (M.B).

The sulfur‐containing compound allicin, known for its antimicrobial and therapeutic properties in garlic, exhibited a consistent decline during storage at both temperature conditions. In Figure 8a, refrigerated storage demonstrated a more effective preservation of allicin content compared to ambient storage (Figure 8b), with (WP.B) and (P‐LDPE) exhibiting slightly higher levels than (LDPE) and (M.B) by day 30. This suggests that regulated oxygen exposure (potentially enabled by semi‐permeable substances such as waxed paper and perforated polyethylene) could permit adequate gaseous exchange to decrease moisture buildup while limiting the oxidative deterioration of allicin.

The observed pattern indicates that allicin degradation may not depend exclusively on moisture retention. Notably, (LDPE), despite its efficacy in reducing water loss, exhibited a marginally accelerated degradation rate. This may result from inadequate oxygen exchange, creating a reductive environment that accelerates the deactivation of alliinase or the volatilization of sulfur compounds. This analysis is consistent with the findings of (Wongsa et al. 2023), which demonstrated that garlic kept in low‐permeability packaging at cold temperatures experienced a quicker depletion of thiosulfates due to oxygen deprivation and internal humidity imbalance.

At 25°C (Figure 8b), there was a rapid and consistent decline in allicin content across all treatments, highlighting the thermal instability of organosulfur compounds. The alignment of degradation curves indicates that ambient temperature not only speeds up enzymatic reactions but also enhances the volatilization of allicin, a compound recognized for its sensitivity to heat. The minimal variations between packaging types highlight the necessity for proactive preservation methods, including oxygen scavengers or sulfur‐stabilizing additives, when garlic is required to be stored at room temperature.

### 3.6 Pyruvic Acid Content Analysis

**FIGURE 9:**
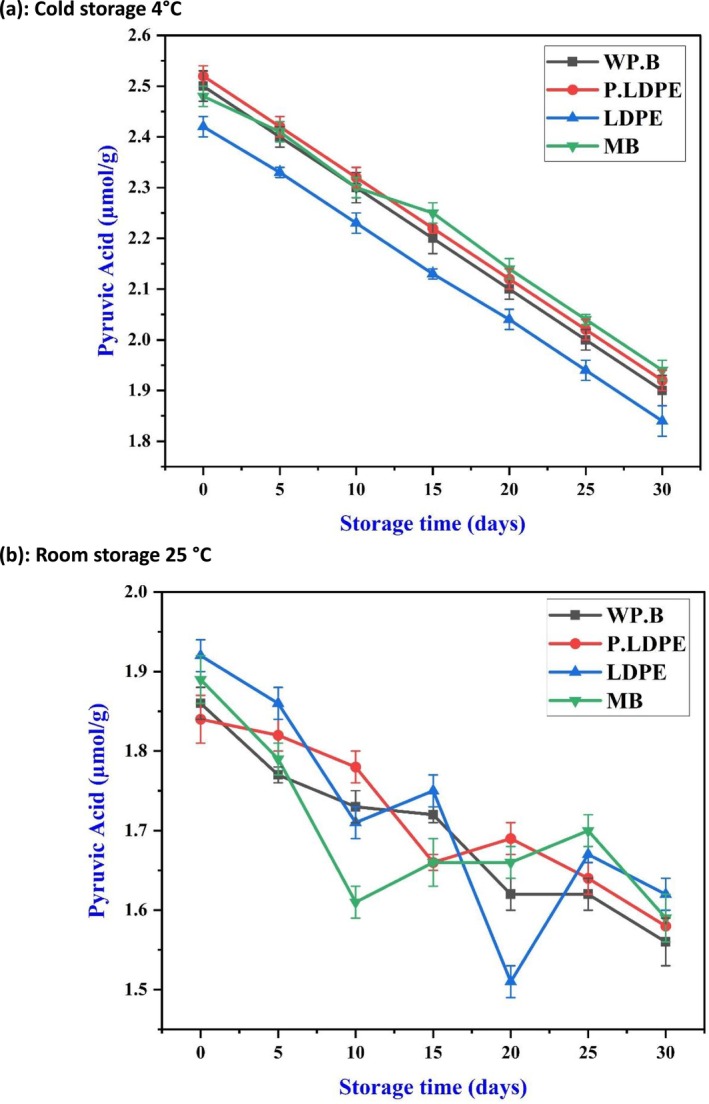
Effects of packaging materials and storage temperatures (a) cold storage 4°C, (b) room storage 25 °C on garlic cloves’ pyruvic acid content. (i) waxed paper bag (WP.B), (ii) low‐density polyethylene (LDPE), (iii) perforated low‐density polyethylene (P‐LDPE), and (iv) mesh bags (M.B).

Figure 9a illustrates that pyruvic acid levels during cold storage demonstrated a steady and uniform decrease across all types of packaging. Interestingly, garlic preserved in (WP.B.) and (P‐LDPE) showed elevated levels of pyruvic acid during the entire storage duration when contrasted with (P‐LDPE) and (LDPE). This finding demonstrates how semi‐permeable packaging can effectively manage oxidative and metabolic stress, which in turn helps to decelerate the enzymatic breakdown of precursors that play a role in the biosynthesis of sulfur compounds. The preservation of pyruvic acid, a byproduct of alliinase activity, is crucial for sustaining the pungency and health benefits associated with garlic, as alliinase is a key enzyme in the formation of allicin and related thiosulfinates. This trend supports the findings of (Eng et al. 2018; González et al. 2013), who observed that garlic kept in low‐temperature, moderate‐humidity environments maintained its enzymatic vitality more effectively than samples exposed to high humidity or oxygen limitation. The findings indicate that waxed paper and perforated polyethylene could provide enhanced microclimatic stability, maintaining alliinase activity by mitigating moisture‐related inactivation or oxygen depletion.

In contrast, ambient storage (Figure 9b) resulted in unpredictable variations and an overall significant decrease in pyruvic acid levels. The different types of packaging showed considerable variation. However, none could sustain stable levels beyond 15 days, highlighting the thermal sensitivity of the enzymatic systems present in garlic. The significant deterioration observed, particularly in samples preserved in (M.B) and (LDPE), indicates that an imbalance of oxygen and the accumulation of moisture in sealed conditions could result in enzyme denaturation or early metabolic exhaustion. The results are consistent with the work of (Sailah et al. 2024) which showed that elevated storage temperatures hasten the oxidative degradation of thiosulfinates and their precursors, making packaging materials significantly less effective in mitigating biochemical losses (Golly et al. 2019; Lv et al. 2019).

### 3.7 Microbiological Analysis

**FIGURE 10:**
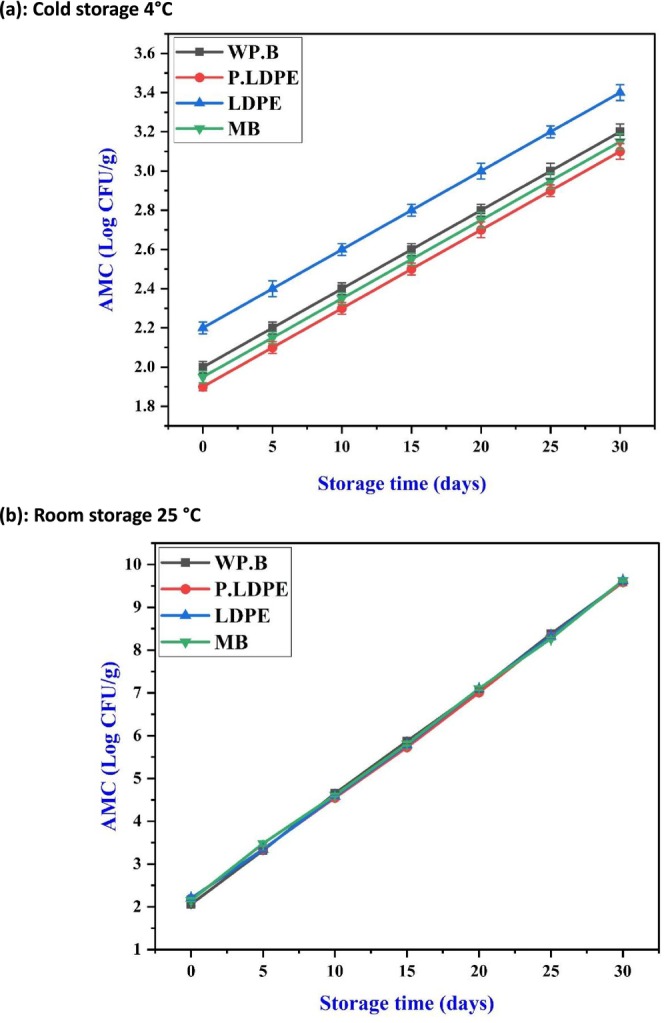
Effects of packaging materials and storage temperatures (a) cold storage 4°C, (b) room storage 25 °C on garlic cloves’ Aerobic Mesophilic Count (AMC). (i) waxed paper bag (WP.B), (ii) low‐density polyethylene (LDPE), (iii) perforated low‐density polyethylene (P‐LDPE), and (iv) mesh bags (M.B).

The Aerobic Mesophilic Count (AMC) demonstrates a gradual increase during refrigerated storage, albeit at a significantly reduced rate when contrasted with ambient conditions. (WP.B) and (P‐LDPE) once again proved to be the most effective packaging options, leading to the lowest microbial proliferation by day 30. The materials in question probably establish a setting conducive to moderate gas exchange, thereby preventing condensation and limiting microbial colonization, particularly from psychrotrophic species that flourish in moisture‐laden environments. Figure 10, illustrating ambient storage, shows a significant and almost linear rise in microbial load across all packaging types, with nearly indistinguishable trajectories. This indicates that temperature is the primary factor influencing microbial growth, rather than the packaging used. The uniformity observed in packaging types indicates that even breathable or low‐permeability films fail to adequately address microbial risk at 25°C, a temperature where rapid cell division and elevated humidity contribute to spoilage. This aligns with earlier observations made by (Sharma and Prasad 2001), who noted comparable patterns in onions and garlic, indicating that microbial counts surged significantly when active preservation methods were not employed.

**FIGURE 11:**
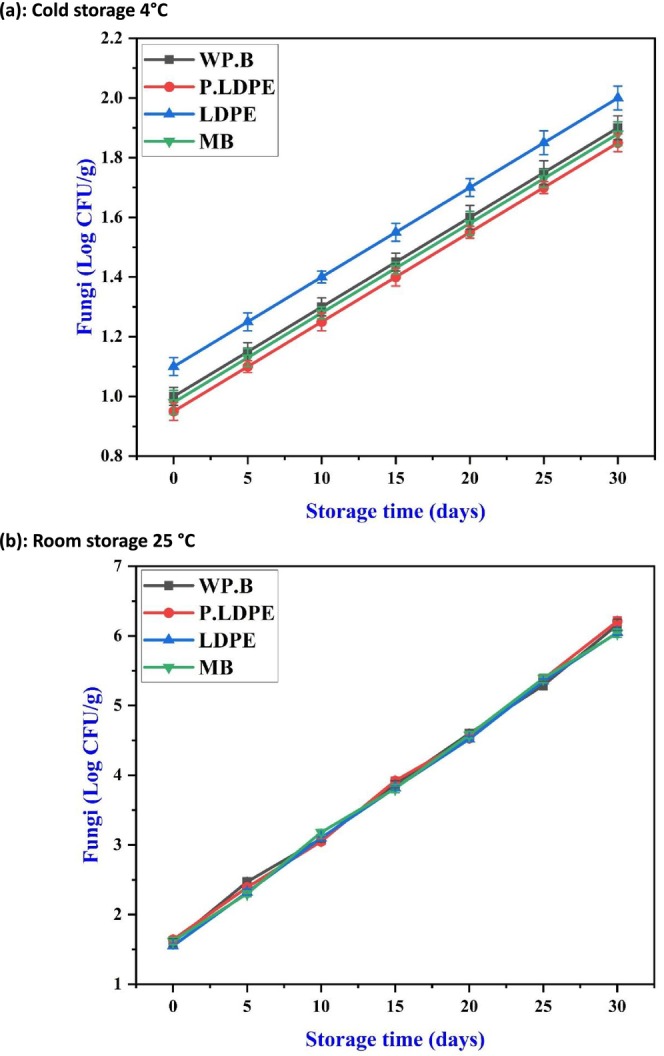
Effects of packaging materials and storage temperatures (a) cold storage 4°C, (b) room storage 25 °C on garlic cloves’ fungal growth. (i) waxed paper bag (WP.B), (ii) low‐density polyethylene (LDPE), (iii) perforated low‐density polyethylene (P‐LDPE), and (iv) mesh bags (M.B).

Under refrigerated conditions (Figure 11a), fungal growth was significantly limited across all packaging types, with only slight increases noted throughout the 30‐day storage period. In the analysis of packaging materials, (WP.B.) and (P‐LDPE) demonstrated the lowest levels of fungal presence, indicating that semi‐permeable barriers facilitate adequate gas exchange while effectively reducing moisture accumulation, which is a recognized factor in the germination of fungal spores. It is essential to highlight that (M.B.) exhibited the poorest performance, probably as a result of its increased porosity and vulnerability to moisture absorption, which fosters microhabitats conducive to mold proliferation. This trend corresponds with findings from (Lanzotti 2006; Zalpouri et al. 2023) who observed that excessive moisture retention in cellulose‐based packaging is associated with increased fungal counts during the storage of root vegetables.

Conversely, Figure 11b demonstrates a swift and almost linear rise in fungal proliferation under ambient conditions, showing minimal variation among packaging types. By day 30, all treatments achieved fungal counts nearing 6.5 Log CFU/g, a significant threshold linked to observable spoilage and the possibility of mycotoxin generation. The results emphasize the significant influence of temperature on fungal control, revealing the limitations of passive packaging in elevated temperature conditions. This pattern reflects the results observed in perishable bulbs as detailed by (González et al. 2013), where storage in ambient conditions without antifungal agents resulted in significant fungal spoilage (Ali et al. 2018; Kwaw et al. 2018).

**FIGURE 12:**
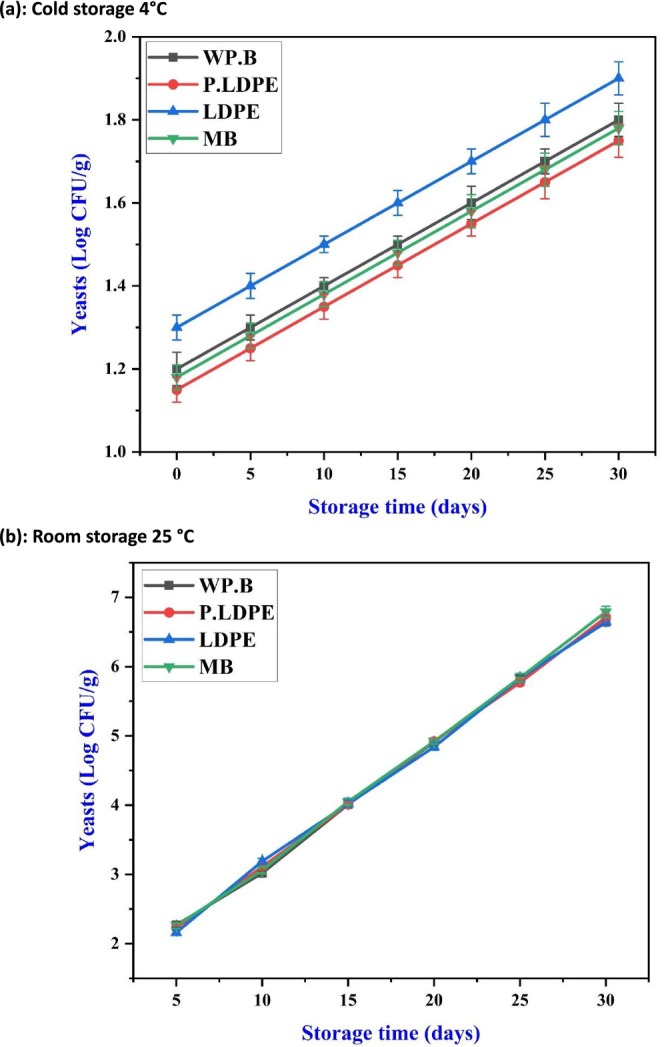
Effects of packaging materials and storage temperatures (a) cold storage 4°C, (b) room storage 25 °C on garlic cloves’ yeast counts. (i) waxed paper bag (WP.B), (ii) low‐density polyethylene (LDPE), (iii) perforated low‐density polyethylene (P‐LDPE), and (iv) mesh bags (M.B).

The dynamics of yeast followed a similar path to that of fungi. At 4°C (Figure 12a), yeast counts exhibited a gradual increase, with (WP.B) and (P‐LDPE) demonstrating slightly better inhibition, particularly during the initial stages. This finding aligns with the hypothesis that moderate levels of oxygen and humidity inhibit the rapid fermentation and biofilm development by yeasts, which can otherwise take advantage of the sugars released during the senescence of garlic. These findings support earlier work by Eng et al. (2018) which emphasized the advantages of maintaining controlled humidity and oxygen levels to inhibit yeast growth in minimally processed vegetables.

In ambient storage (Figure 12b), yeast counts increased consistently and uniformly, with no notable benefit observed from any of the packaging types. The convergence of all lines suggests a consistent colonization pattern, highlighting the remarkable adaptability of yeasts to aerobic, nutrient‐rich, and warm environments. The absence of packaging differentiation indicates that only active solutions, like those with fungistatic or yeast‐inhibiting compounds, may offer effective protection in non‐refrigerated conditions (Rashid et al. 2018; Zhou et al. 2018).

### 3.8 Statistical Interpretation of the Gallic's Physicochemical Properties

#### 3.8.1 Principal Component Analysis (PCA)

**FIGURE 13:**
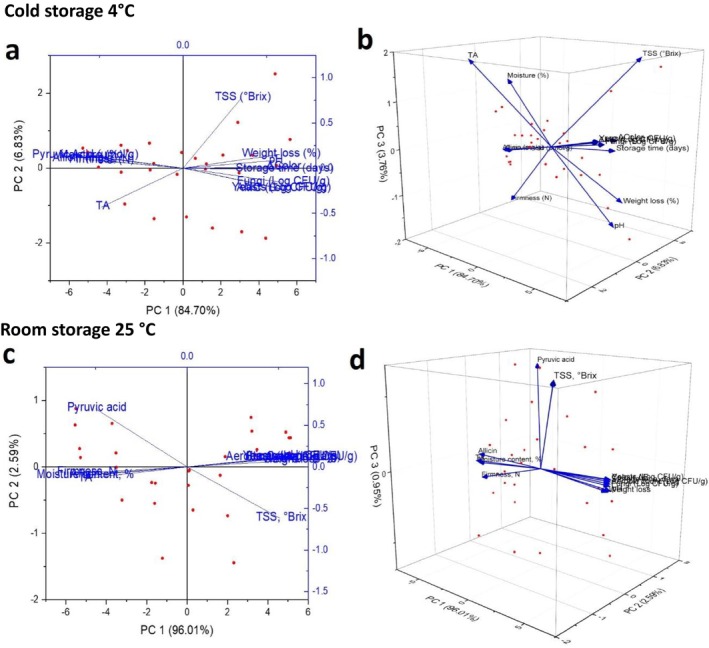
Three‐dimensional biplot ordination on principal component analysis of the storage process (a) 2D PCA biplot, and (b) 3D biplot.

The principal component analysis (PCA) illustrated in Figure 13 provides a comprehensive view of the interactions between physicochemical and microbiological quality changes in garlic stored at two distinct temperatures (4°C and 25°C), complementing and expanding upon earlier research in garlic and allium postharvest studies. At 4°C (Figure 13a,b), PCA indicates that PC1 explains 84.70% of the total variation, distinctly highlighting trends in quality deterioration. Characteristics like TSS (°Brix), weight loss, fungal and yeast counts, and storage duration are closely grouped along the positive PC1 axis. This classification indicates that refrigerated storage continues to encourage mild metabolic activity and microbial growth over time, albeit at a reduced pace in comparison to ambient conditions. In the course of storage, it is observed that traits such as titratable acidity (TA), pyruvic acid, and moisture exhibit a negative correlation with PC1. This suggests that as storage time extends, there is an increase in sugar content, while acidity, moisture, and pungency tend to decrease. This aligns with earlier investigations (González et al. 2013; Ludlow et al. 2021) that demonstrated how low‐temperature storage results in gradual yet quantifiable biochemical alterations, particularly the degradation of pungency indicators like pyruvic acid and alliin. Interestingly, the PCA conducted at 25°C (Figure 13c,d) reveals a distinctly deterministic decay trajectory, with PC1 accounting for an impressive 96.01% of the total variance. In this context, the decline in quality is primarily influenced by swift decreases in moisture, firmness, and pyruvic acid, coupled with significant rises in TSS and microbial counts. This trend aligns with previous findings by (El‐Mesery, Qenawy, et al. 2024; Madhu et al. 2019), who showed that alliums kept at ambient temperatures experience increased enzymatic activity and microbial degradation, influenced by elevated metabolic rates. The clustering of aerobic mesophilic counts (AMC), fungal and yeast growth, and TSS within the same quadrant demonstrates a closely linked spoilage process that prevails in non‐refrigerated environments. To ensure clarity, the PCA loading values are provided in Tables S1 and S2, which allow direct interpretation of variable contributions.

#### 3.8.2 Correlation Analysis

The correlation matrix for garlic stored at 4°C offers essential insights into the dynamics of quality degradation over time, consistent with the PCA analysis findings. The most significant positive correlations with storage time are seen in weight loss (*r* = 0.943), ∆Color (*r* = 0.996), and fungal growth (*r* = 0.983), suggesting that both visual and microbial spoilage develop consistently during refrigeration (Table 1). It is important to note that microbial parameters, including total aerobic mesophilic counts (AMC, *r* = 0.962), fungi (*r* = 0.983), and yeasts (*r* = 0.963), exhibit consistent and robust positive correlations with time. This underscores that microbial activity continues to be a significant issue, even at lower temperatures. Conversely, essential indicators of freshness such as firmness (*r* = −0.822), moisture (*r* = −0.899), allicin content (*r* = −0.991), and pyruvic acid (*r* = −0.986), exhibit a negative correlation with storage duration. The significant decreases highlight the deterioration of structural and biochemical integrity over the storage period. Allicin and pyruvic acid, both bioactive sulfur compounds, exhibit nearly perfect negative correlations with time and microbial load, supporting earlier studies (González et al. 2013; Gryzenhout et al. 2021) that highlighted their degradation as a spoilage marker and their function in natural antimicrobial defense. The robust inter‐correlation observed among spoilage markers (fungi and yeasts: *r* = 0.996) and the negative correlations with quality traits (pyruvic acid vs. fungi: *r* = −0.999) further substantiate the PCA findings, indicating that these variables are closely clustered and change in contrasting directions throughout storage. The multivariate coherence observed indicates that refrigerated garlic experiences a systematic and foreseeable decline in quality, aligning with previous studies (Kalathingal et al. 2020; Zadhossein et al. 2023), and highlights the importance of comprehensive monitoring in postharvest management.

**TABLE 1:** Matrix of correlations of garlic storage (4°C).

Variables	(1)	(2)	(3)	(4)	(5)	(6)	(7)	(8)	(9)	(10)	(11)	(12)	(13)
(1) Storagetimedays	1.000												
(2) Firmnessn	−0.822	1.000											
(3) Weightloss	0.943	−0.763	1.000										
(4) TSSBrix	0.575	−0.455	0.596	1.000									
(5) ta	−0.786	0.594	−0.890	−0.628	1.000								
(6) ph	0.904	−0.643	0.928	0.513	−0.793	1.000							
(7) Moisture	−0.899	0.760	−0.923	−0.381	0.768	−0.875	1.000						
(8) Î″Color	0.996	−0.820	0.933	0.601	−0.781	0.888	−0.875	1.000					
(9) allicinmgg	−0.991	0.805	−0.917	−0.533	0.737	−0.871	0.901	−0.987	1.000				
(10) PA	−0.986	0.800	−0.905	−0.508	0.714	−0.864	0.898	−0.980	0.998	1.000			
(11) amclogcfug	0.962	−0.775	0.869	0.442	−0.662	0.828	−0.896	0.951	−0.987	−0.993	1.000		
(12) fungilogcfug	0.983	−0.797	0.901	0.492	−0.708	0.859	−0.903	0.975	−0.997	−0.999	0.996	1.000	
(13) yeastslogcfug	0.963	−0.775	0.869	0.446	−0.663	0.827	−0.894	0.952	−0.988	−0.993	1.000	0.996	1.000

The correlation matrix for garlic stored at 25°C indicates a more significant and rapid decline in quality when compared to cold storage, highlighting the increased metabolic and microbial activities present at room temperature (Table 2). The duration of storage exhibits robust positive correlations with weight loss (*r* = 0.995), total color change (*r* = 1.000), and microbial counts (AMC) (*r* = 1.000), fungi (*r* = 0.999), and yeasts (*r* = 1.000). The observed values suggest a rapid and straightforward advancement of spoilage, aligning with the PCA findings that grouped microbial and physicochemical degradation factors closely along PC1. The analysis reveals a significant negative correlation between firmness and time (*r* = −0.981), highlighting the swift softening associated with cellular degradation. Allicin (*r* = −0.999) and moisture content (*r* = −0.996) demonstrate remarkably high negative correlations with time, indicating a significant decline in bioactive and hydration‐related properties. This finding aligns with earlier research (Rai et al. 2022; Sarkar et al. 2023), which shows that heat accelerates the degradation of sulfur compounds in garlic. Similarly, the levels of pyruvic acid also show a significant decline (*r* = −0.857), albeit at a slightly less steep rate compared to cold storage, which may be attributed to enzymatic volatility at elevated temperatures. It is noteworthy that total soluble solids (TSS) exhibit a significant positive correlation with storage time (*r* = 0.872), indicating an increase in sugar concentration as a result of moisture loss. The inverse correlation of titratable acidity (TA) with time (*r* = −0.999) underscores the diminishing freshness. The relative warm storage profile is consistent with previous findings by Salehi and Kashaninejad (2018); Tuly et al. (2023) and emphasizes that storing at room temperature significantly hastens spoilage due to combined physical, chemical, and microbial changes, highlighting the need for rigorous cold chain practices for garlic preservation.

**TABLE 2:** Correlation matrix of garlic storage (25°C).

Variables	(1)	(2)	(3)	(4)	(5)	(6)	(7)	(8)	(9)	(10)	(11)	(12)	(13)
(1) Storage time (days)	1.000												
(2) Firmness	−0.981	1.000											
(3) Weight loss	0.995	−0.976	1.000										
(4) TSS	0.872	−0.876	0.870	1.000									
(5) TA	−0.999	0.981	−0.993	−0.865	1.000								
(6) pH	0.997	−0.973	0.990	0.860	−0.996	1.000							
(7) Moisture content	−0.996	0.976	−0.991	−0.873	0.995	−0.993	1.000						
(8) Color	1.000	−0.980	0.994	0.873	−0.999	0.997	−0.996	1.000					
(9) Allicin	−0.999	0.980	−0.995	−0.872	0.998	−0.997	0.995	−0.999	1.000				
(10) PA	−0.857	0.842	−0.867	−0.880	0.846	−0.856	0.851	−0.856	0.861	1.000			
(11) AMC	1.000	−0.980	0.995	0.871	−0.999	0.997	−0.996	1.000	−0.999	−0.858	1.000		
(12) Fungi	0.999	−0.977	0.994	0.869	−0.998	0.997	−0.995	0.999	−0.998	−0.859	0.999	1.000	
(13) Yeasts	1.000	−0.981	0.995	0.872	−0.999	0.996	−0.996	0.999	−0.999	−0.854	0.999	0.999	1.000

#### 3.8.3 One‐Way ANOVA Analysis

The one‐way ANOVA results indicated that under refrigerated storage (4°C), no statistically significant differences (*p* > 0.05) were detected among packaging materials for firmness, weight loss, allicin, pyruvic acid, or microbial counts. However, waxed paper and perforated polyethylene showed slightly better preservation trends compared to polyethylene and paper bags (Table S3). In contrast, at 25°C, packaging type had no significant effect on any measured parameter, and all treatments underwent rapid and uniform deterioration (Table S4). These findings confirm that storage temperature is the critical factor influencing garlic shelf‐life, while the contribution of packaging is comparatively minor.

## 4 Conclusion

This investigation rigorously assessed the impact of packaging materials and storage temperatures on garlic's physicochemical, phytochemical, and microbiological stability ( *Allium sativum* *L*.) throughout a 30‐day duration. The findings indicate that temperature is the main factor influencing quality decline, with storage at ambient conditions (25°C) notably hastening the reduction in firmness, moisture, allicin, and pyruvic acid, while encouraging rapid microbial proliferation. In contrast, refrigerated storage at 4°C significantly reduced physicochemical and microbiological spoilage, especially when garlic was packaged in waxed paper or perforated polyethylene. The materials established a well‐regulated microenvironment that reduced moisture loss, moderated respiration‐driven biochemical degradation, and limited microbial growth. The results provide compelling evidence for the incorporation of sophisticated modeling techniques in the study of garlic storage. The dynamics of color change can be accurately characterized through zero‐ or first‐order models, facilitating the predictive monitoring of visual quality. Similarly, thermodynamic models of the Arrhenius type could be utilized to analyze the degradation of allicin, enabling predictions regarding its biochemical stability throughout various storage conditions. Simultaneously, spectroscopic fingerprinting can uncover further phytochemical changes that traditional metrics may overlook. Furthermore, the correlation and PCA findings validated that microbial spoilage is closely associated with reductions in moisture, pyruvic acid, and bioactive compounds, establishing a comprehensive spoilage model appropriate for predictive quality control. Subsequent investigations should focus on advancing intelligent packaging technologies, including moisture‐regulating films, stabilizers for volatile compounds, and antifungal coatings enhanced with natural agents such as thymol or eugenol.

## Author Contributions

**Hany S. El‐Mesery:** conceptualization (equal), data curation (equal), formal analysis (equal), funding acquisition (equal), investigation (equal), methodology (equal), resources (equal), software (equal), supervision (equal), validation (equal), visualization (equal), writing – original draft (equal), writing – review and editing (equal). **Mansuur Husein:** data curation (equal), formal analysis (equal), writing – original draft (equal), writing – review and editing (equal). **Zicheng Hu:** investigation (equal), resources (equal), validation (equal). **Mona A. Elabd:** data curation (equal), formal analysis (equal), methodology (equal).

## Conflicts of Interest

The authors declare no conflicts of interest.

## Supporting information


**Table S1:** fsn371170‐sup‐0001‐TableS1‐S4.docx.

## Data Availability

The original contributions presented in the study are included in the article; further inquiries can be directed to the first author (Hany S. El‐Mesery, elmesiry@ujs.edu.cn) and the corresponding author.
