# Supplementary material for: Postharvest Stability of Peeled Garlic Cloves (Allium sativum L.): A Study on Packaging Materials and Shelf‐Life Environmental Interactions
Source: Food Sci Nutr. 2025 Nov 10;13(11):e71170. doi: 10.1002/fsn3.71170 (PMC12598304; doi:10.1002/fsn3.71170)
Supplement: Supplementary file 1 — Table S1: fsn371170‐sup‐0001‐TableS1‐S4.docx. [file FSN3-13-e71170-s001.docx]

TABLE S1 PCA Loadings (4 °C)

| Principal Component Number | Eigenvalues | | PC 1 | PC 2 | PC 3 | PC 1 | PC 2 | PC 3 |
| --- | --- | --- | --- | --- | --- | --- | --- | --- |
|  |  |  | 84.70% | 6.83% | 3.76% | 84.70% | 6.83% | 3.76% |
| Eigenvalue | Eigenvalue | Loading Plot | Loading Plot | Loading Plot | Loading Plot | Loading Plot | Loading Plot | Loading Plot |
| 1 | 11.01117 | Storage time (days) | 0 | 0 | 0 | 5.63525 | -0.04674 | 0.19506 |
| 2 | 0.88783 | Firmness (N) | 0 | 0 | 0 | -4.66903 | 0.27728 | -1.5555 |
| 3 | 0.48822 | Weight loss (%) | 0 | 0 | 0 | 5.43714 | 0.49107 | -1.00578 |
| 4 | 0.3358 | TSS (°Brix) | 0 | 0 | 0 | 3.28435 | 2.51351 | 1.80095 |
| 5 | 0.14272 | TA | 0 | 0 | 0 | -4.58132 | -1.39416 | 1.83825 |
| 6 | 0.09291 | pH | 0 | 0 | 0 | 5.14727 | 0.27307 | -1.52016 |
| 7 | 0.02414 | Moisture (%) | 0 | 0 | 0 | -5.25836 | 0.44388 | 1.25818 |
| 8 | 0.01444 | ΔColor | 0 | 0 | 0 | 5.59627 | 0.04902 | 0.40568 |
| 9 | 0.00196 | Allicin (mg/g) | 0 | 0 | 0 | -5.60175 | 0.32279 | -0.38921 |
| 10 | 6.41E-04 | Pyruvic Acid (µmol/g) | 0 | 0 | 0 | -5.57264 | 0.44638 | -0.42551 |
| 11 | 1.66E-04 | AMC (Log CFU/g) | 0 | 0 | 0 | 5.45364 | -0.73672 | 0.45297 |
| 12 | 7.26E-07 | Fungi (Log CFU/g) | 0 | 0 | 0 | 5.56016 | -0.50894 | 0.38849 |
| 13 | 4.55E-32 | Yeasts (Log CFU/g) | 0 | 0 | 0 | 5.45617 | -0.72519 | 0.47685 |

Table S2 PCA Loadings (25 °C)

| Principal Component Number | Eigenvalues | | PC 1 | PC 2 | PC 3 | PC 1 | PC 2 | PC 3 |
| --- | --- | --- | --- | --- | --- | --- | --- | --- |
|  |  |  | 96.01% | 2.59% | 0.95% | 96.01% | 2.59% | 0.95% |
| Eigenvalue | Eigenvalue | Loading Plot | Loading Plot | Loading Plot | Loading Plot | Loading Plot | Loading Plot | Loading Plot |
| 1 | 12.48079 | Storage time, days | 0 | 0 | 0 | 5.54607 | 0.22586 | -0.00975 |
| 2 | 0.33708 | Firmness, N | 0 | 0 | 0 | -5.46207 | -0.14906 | -0.14691 |
| 3 | 0.1233 | Weight loss | 0 | 0 | 0 | 5.52848 | 0.15809 | -0.07406 |
| 4 | 0.034 | TSS, °Brix | 0 | 0 | 0 | 4.98923 | -1.2754 | 0.69432 |
| 5 | 0.00991 | TA | 0 | 0 | 0 | -5.53497 | -0.29544 | -0.01139 |
| 6 | 0.00729 | pH | 0 | 0 | 0 | 5.52707 | 0.2587 | -0.07663 |
| 7 | 0.00315 | Moisture content, % | 0 | 0 | 0 | -5.52734 | -0.22421 | -0.03006 |
| 8 | 0.00187 | Colour ΔE | 0 | 0 | 0 | 5.54517 | 0.2279 | 0.00111 |
| 9 | 0.00127 | Allicin | 0 | 0 | 0 | -5.54529 | -0.20413 | 0.03111 |
| 10 | 6.36E-04 | Pyruvic acid | 0 | 0 | 0 | -4.91472 | 1.44285 | 0.62899 |
| 11 | 5.30E-04 | Aerobic count (Log CFU/g) | 0 | 0 | 0 | 5.54506 | 0.2254 | -0.02535 |
| 12 | 1.30E-04 | Fungi (Log CFU/g) | 0 | 0 | 0 | 5.5416 | 0.22388 | -0.0411 |
| 13 | 4.24E-05 | Yeasts (Log CFU/g) | 0 | 0 | 0 | 5.54359 | 0.23867 | 0.00312 |

**Table S3 Pairwise correlations** (4 °C)

| Variables |  | (2) | (3) | (4) | (5) | (6) | (7) | (8) | (9) | (10) | (11) | (12) | (13) |  |
| --- | --- | --- | --- | --- | --- | --- | --- | --- | --- | --- | --- | --- | --- | --- |
| (1) storagetimedays | 1.000 |  |  |  |  |  |  |  |  |  |  |  |  |  |
|  |  |  |  |  |  |  |  |  |  |  |  |  |  |  |
| (2) firmnessn | -0.822 | 1.000 |  |  |  |  |  |  |  |  |  |  |  |  |
|  | (0.000*) |  |  |  |  |  |  |  |  |  |  |  |  |  |
| (3) weightloss | 0.943 | -0.763 | 1.000 |  |  |  |  |  |  |  |  |  |  |  |
|  | (0.000*) | (0.000*) |  |  |  |  |  |  |  |  |  |  |  |  |
| (4) TSSBrix | 0.575 | -0.455 | 0.596 | 1.000 |  |  |  |  |  |  |  |  |  |  |
|  | (0.001*) | (0.015*) | (0.001*) |  |  |  |  |  |  |  |  |  |  |  |
| (5) ta | -0.786 | 0.594 | -0.890 | -0.628 | 1.000 |  |  |  |  |  |  |  |  |  |
|  | (0.000*) | (0.001*) | (0.000*) | (0.000*) |  |  |  |  |  |  |  |  |  |  |
| (6) ph | 0.904 | -0.643 | 0.928 | 0.513 | -0.793 | 1.000 |  |  |  |  |  |  |  |  |
|  | (0.000*) | (0.000*) | (0.000*) | (0.005*) | (0.000*) |  |  |  |  |  |  |  |  |  |
| (7) moisture | -0.899 | 0.760 | -0.923 | -0.381 | 0.768 | -0.875 | 1.000 |  |  |  |  |  |  |  |
|  | (0.000*) | (0.000*) | (0.000*) | (0.046*) | (0.000*) | (0.000*) |  |  |  |  |  |  |  |  |
| (8) Î”Color | 0.996 | -0.820 | 0.933 | 0.601 | -0.781 | 0.888 | -0.875 | 1.000 |  |  |  |  |  |  |
|  | (0.000*) | (0.000*) | (0.000*) | (0.001*) | (0.000*) | (0.000*) | (0.000*) |  |  |  |  |  |  |  |
| (9) allicinmgg | -0.991 | 0.805 | -0.917 | -0.533 | 0.737 | -0.871 | 0.901 | -0.987 | 1.000 |  |  |  |  |  |
|  | (0.000*) | (0.000*) | (0.000*) | (0.004*) | (0.000*) | (0.000*) | (0.000*) | (0.000*) |  |  |  |  |  |  |
| (10) PyruvicAcidÂµm~g | -0.986 | 0.800 | -0.905 | -0.508 | 0.714 | -0.864 | 0.898 | -0.980 | 0.998 | 1.000 |  |  |  |  |
|  | (0.000*) | (0.000*) | (0.000*) | (0.006*) | (0.000*) | (0.000*) | (0.000*) | (0.000*) | (0.000*) |  |  |  |  |  |
| (11) amclogcfug | 0.962 | -0.775 | 0.869 | 0.442 | -0.662 | 0.828 | -0.896 | 0.951 | -0.987 | -0.993 | 1.000 |  |  |  |
|  | (0.000*) | (0.000*) | (0.000*) | (0.018*) | (0.000*) | (0.000*) | (0.000*) | (0.000*) | (0.000*) | (0.000*) |  |  |  |  |
| (12) fungilogcfug | 0.983 | -0.797 | 0.901 | 0.492 | -0.708 | 0.859 | -0.903 | 0.975 | -0.997 | -0.999 | 0.996 | 1.000 |  |  |
|  | (0.000*) | (0.000*) | (0.000*) | (0.008*) | (0.000*) | (0.000*) | (0.000*) | (0.000*) | (0.000*) | (0.000*) | (0.000*) |  |  |  |
| (13) yeastslogcfug | 0.963 | -0.775 | 0.869 | 0.446 | -0.663 | 0.827 | -0.894 | 0.952 | -0.988 | -0.993 | 1.000 | 0.996 | 1.000 |  |
|  | (0.000*) | (0.000*) | (0.000*) | (0.017*) | (0.000*) | (0.000*) | (0.000*) | (0.000*) | (0.000*) | (0.000*) | (0.000*) | (0.000*) |  |  |
|  |  |  |  |  |  |  |  |  |  |  |  |  |  |  |
|  |  |  |  |  |  |  |  |  |  |  |  |  |  |  |
|  | | | | | | | | | | | | | | |

**Table S4 Pairwise correlations (25**°C)

| Variables |  | (2) | (3) | (4) | (5) | (6) | (7) | (8) | (9) | (10) | (11) | (12) | (13) |  |
| --- | --- | --- | --- | --- | --- | --- | --- | --- | --- | --- | --- | --- | --- | --- |
| (1) storagetimedays | 1.000 |  |  |  |  |  |  |  |  |  |  |  |  |  |
|  |  |  |  |  |  |  |  |  |  |  |  |  |  |  |
| (2) firmnessn | -0.981 | 1.000 |  |  |  |  |  |  |  |  |  |  |  |  |
|  | (0.000*) |  |  |  |  |  |  |  |  |  |  |  |  |  |
| (3) weightloss | 0.995 | -0.976 | 1.000 |  |  |  |  |  |  |  |  |  |  |  |
|  | (0.000*) | (0.000*) |  |  |  |  |  |  |  |  |  |  |  |  |
| (4) TSSBrix | 0.872 | -0.876 | 0.870 | 1.000 |  |  |  |  |  |  |  |  |  |  |
|  | (0.000*) | (0.000*) | (0.000*) |  |  |  |  |  |  |  |  |  |  |  |
| (5) ta | -0.999 | 0.981 | -0.993 | -0.865 | 1.000 |  |  |  |  |  |  |  |  |  |
|  | (0.000*) | (0.000*) | (0.000*) | (0.000*) |  |  |  |  |  |  |  |  |  |  |
| (6) ph | 0.997 | -0.973 | 0.990 | 0.860 | -0.996 | 1.000 |  |  |  |  |  |  |  |  |
|  | (0.000*) | (0.000*) | (0.000*) | (0.000*) | (0.000*) |  |  |  |  |  |  |  |  |  |
| (7) moisturecontent | -0.996 | 0.976 | -0.991 | -0.873 | 0.995 | -0.993 | 1.000 |  |  |  |  |  |  |  |
|  | (0.000*) | (0.000*) | (0.000*) | (0.000*) | (0.000*) | (0.000*) |  |  |  |  |  |  |  |  |
| (8) ColourÎ”E | 1.000 | -0.980 | 0.994 | 0.873 | -0.999 | 0.997 | -0.996 | 1.000 |  |  |  |  |  |  |
|  | (0.000*) | (0.000*) | (0.000*) | (0.000*) | (0.000*) | (0.000*) | (0.000*) |  |  |  |  |  |  |  |
| (9) allicin | -0.999 | 0.980 | -0.995 | -0.872 | 0.998 | -0.997 | 0.995 | -0.999 | 1.000 |  |  |  |  |  |
|  | (0.000*) | (0.000*) | (0.000*) | (0.000*) | (0.000*) | (0.000*) | (0.000*) | (0.000*) |  |  |  |  |  |  |
| (10) pyruvicacid | -0.857 | 0.842 | -0.867 | -0.880 | 0.846 | -0.856 | 0.851 | -0.856 | 0.861 | 1.000 |  |  |  |  |
|  | (0.000*) | (0.000*) | (0.000*) | (0.000*) | (0.000*) | (0.000*) | (0.000*) | (0.000*) | (0.000*) |  |  |  |  |  |
| (11) aerobiccountl~g | 1.000 | -0.980 | 0.995 | 0.871 | -0.999 | 0.997 | -0.996 | 1.000 | -0.999 | -0.858 | 1.000 |  |  |  |
|  | (0.000*) | (0.000*) | (0.000*) | (0.000*) | (0.000*) | (0.000*) | (0.000*) | (0.000*) | (0.000*) | (0.000*) |  |  |  |  |
| (12) fungilogcfug | 0.999 | -0.977 | 0.994 | 0.869 | -0.998 | 0.997 | -0.995 | 0.999 | -0.998 | -0.859 | 0.999 | 1.000 |  |  |
|  | (0.000*) | (0.000*) | (0.000*) | (0.000*) | (0.000*) | (0.000*) | (0.000*) | (0.000*) | (0.000*) | (0.000*) | (0.000*) |  |  |  |
| (13) yeastslogcfug | 1.000 | -0.981 | 0.995 | 0.872 | -0.999 | 0.996 | -0.996 | 0.999 | -0.999 | -0.854 | 0.999 | 0.999 | 1.000 |  |
|  | (0.000*) | (0.000*) | (0.000*) | (0.000*) | (0.000*) | (0.000*) | (0.000*) | (0.000*) | (0.000*) | (0.000*) | (0.000*) | (0.000*) |  |  |
|  |  |  |  |  |  |  |  |  |  |  |  |  |  |  |
|  |  |  |  |  |  |  |  |  |  |  |  |  |  |  |
|  | | | | | | | | | | | | | | |

**One-Way ANOVA Analysis**

The one-way ANOVA results under refrigerated storage at 4 °C revealed no statistically significant differences among packaging treatments for most physicochemical, biochemical, and microbiological parameters (p > 0.05). Firmness, weight loss, moisture content, color change (ΔE), allicin, pyruvic acid, and microbial counts (aerobic mesophilic, fungi, and yeasts) all exhibited comparable mean values across packaging types, indicating that low temperature was the dominant factor in maintaining garlic quality. Although minor numerical variations were observed. Materials such as waxed paper and perforated polyethylene tended to retain slightly higher firmness and pyruvic acid levels, while high-density polyethylene minimized weight loss, these differences were not statistically significant. Titratable acidity and TSS showed trends toward variability, but again did not reach significance (p = 0.092–0.094). These findings demonstrate that at 4 °C, cold storage alone effectively suppressed physiological deterioration and microbial proliferation, diminishing the relative influence of packaging materials on garlic quality preservation.

Similarly, the ANOVA results for garlic stored at 25 °C demonstrated that none of the measured parameters differed significantly among the packaging treatments (p > 0.05). Variables including firmness, weight loss, TSS, titratable acidity, pH, moisture content, color change (ΔE), allicin, pyruvic acid, and microbial counts (aerobic mesophilic bacteria, fungi, and yeasts) all showed statistically similar mean values across waxed paper, perforated polyethylene, polyethylene, and paper bags. Despite this lack of statistical differentiation, the descriptive data confirmed that all treatments experienced substantial deterioration during storage at ambient temperature, with marked reductions in firmness, allicin, and pyruvic acid and corresponding increases in microbial populations and weight loss. These findings highlight that elevated temperature is the overriding factor driving garlic quality decline, overwhelming the moderating effects of packaging. In contrast to cold storage, where packaging exerted modest influence, ambient storage conditions led to rapid and uniform degradation, reinforcing the necessity of refrigeration to extend shelf life and preserve garlic’s physicochemical and phytochemical integrity.

The one-way ANOVA results indicated that under refrigerated storage (4 °C), no statistically significant differences (p > 0.05) were detected among packaging materials for firmness, weight loss, allicin, pyruvic acid, or microbial counts, although waxed paper and perforated polyethylene showed slightly better preservation trends compared to polyethylene and paper bags. In contrast, at 25 °C, packaging type had no significant effect on any measured parameter, and all treatments underwent rapid and uniform deterioration. These findings confirm that storage temperature is the critical factor influencing garlic shelf-life, while the contribution of packaging is comparatively minor.
